# Endogenous endophthalmitis caused by urinary tract infection: A case report

**DOI:** 10.1097/MD.0000000000036139

**Published:** 2023-11-17

**Authors:** Cong Ren, Zhongen Li, Fan Meng, Yongle Du, Hao Sun, Bin Guo

**Affiliations:** a Shandong University of Traditional Chinese Medicine, Jinan, China; b Eye Hospital Affiliated to Shandong University of Traditional Chinese Medicine, Jinan, China; c Shandong Academy of Eye Disease Prevention and Therapy/Shandong Provincial Key Laboratory of Integrated Traditional Chinese and Western Medicine for Prevention and Treatment of Eye Disease, Jinan, China; d Department of Ophthalmology, The Fourth Affiliated Hospital of China Medical University, Eye Hospital of China Medical University, Shenyang, China; e Lanling People’s Hospital of Linyi City, Linyi, China; f Postdoctoral Station of Shandong University of Traditional Chinese Medicine, Jinan, China.

**Keywords:** case report, endogenous endophthalmitis, *Klebsiella pneumonia*, risk factor, urinary tract infection

## Abstract

**Rationale:**

Endogenous endophthalmitis is a vision-threatening intraocular infection caused by hematogenous spread of infectious organisms from distant sites.

**Patient concerns:**

A 71-year-old man with a history of fever and dysuria 5 days prior to presentation presented with sudden loss of vision in his left eye. The patient had no history of ocular surgery or trauma, and ocular examination revealed a large amount of exudative plaque covering the pupil. Therefore, fundus examination was not feasible. B-scan ultrasonography revealed a dome-shaped subretinal mass with an exudative retinal detachment.

**Diagnosis:**

Endogenous endophthalmitis was diagnosed on the basis of these findings.

**Interventions:**

The patient underwent pars plana vitrectomy and the early postoperative course was favorable.

**Outcomes:**

Vitreous cultures grew gram-negative bacilli, identified as *Klebsiella pneumonia*. Urinalysis revealed white blood cells (++) and urinary tract infection was the only identifiable risk factor for endogenous endophthalmitis.

**Lessons:**

Urinary tract infection is an independent risk factor for endogenous endophthalmitis.

## 1. Introduction

Endogenous endophthalmitis is a vision-threatening intraocular infection caused by the hematogenous spread of infectious organisms from distant sites into the eye.^[1]^ Approximately 2% to 8% of endophthalmitis cases are associated with endogenous sources, and the most common causative organism is *Staphylococcus aureus*.^[2–4]^ In Southeast Asia, Klebsiella pneumoniae (KP), which is often associated with a pyogenic liver abscesses (LA) and diabetes mellitus, is an important cause of endogenous endophthalmitis (3–37%).^[5]^ Herein, we report the case of a 71-year-old man with a history of fever and dysuria who developed endogenous endophthalmitis. Additionally, we reviewed the literature on this subject.

## 2. Case presentation

**Figure 1.**
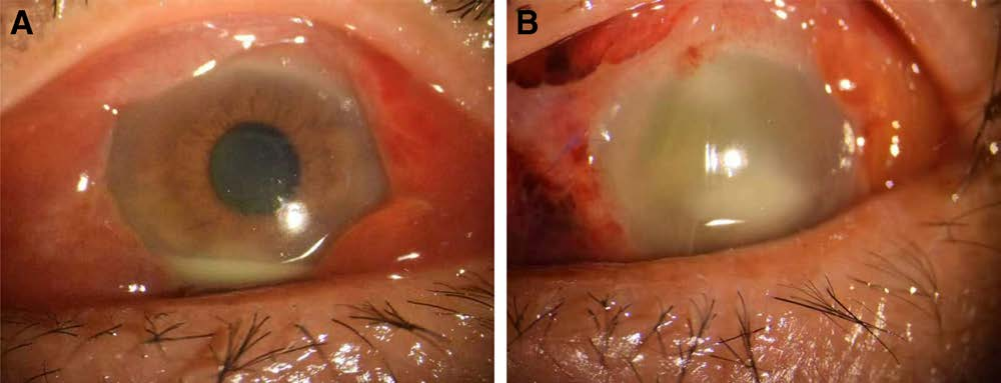
(A) The conjunctiva was congested, highly edema, mild corneal edema, pyEMA in the anterior chamber, pupil exudation, and exudation into the fundus. (B) The vitreous cavity was filled with silicone oil. When patient lowered his head, the corneal edema was more severe than before, the anterior chamber empyema, the remaining peep out.

A 71-year-old man presented with a two-day history of redness and sudden vision loss in his left eye. The best-corrected visual acuity was hand motion, and a noncontact tonometer revealed an intraocular pressure of 17 mm Hg in the left eye. Upon examination, the right eye showed no obvious abnormalities; however, lid edema, conjunctival chemosis, corneal edema, and a 2-mm hypopyon were observed in the left eye (Fig. 1A). Exudates were observed adhering to the posterior surface of the lens and filling the vitreous cavity through the pupillary area, making fundus examination infeasible.

**Figure 2.**
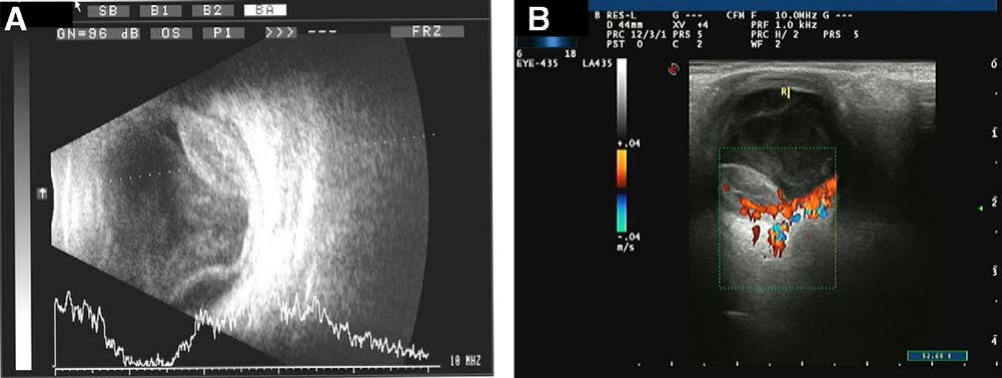
B-scan (A) and color ultrasound (B) revealed a dome-shaped subretinal mass with exudative retinal detachment.

**Figure 3.**
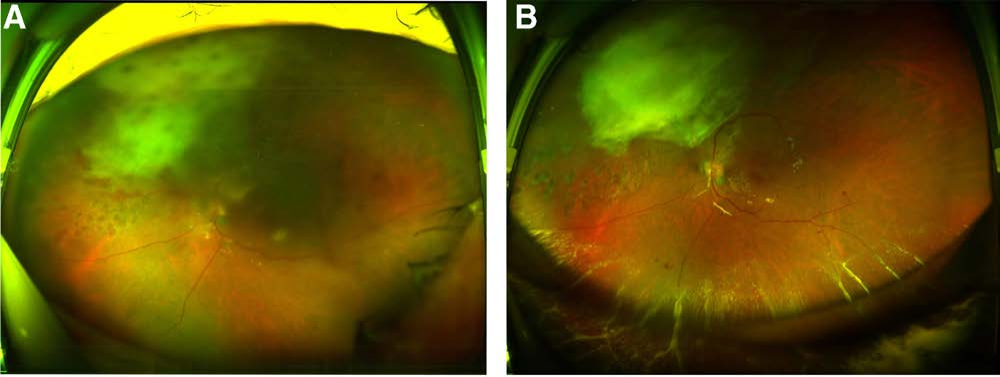
Gray-white area for the original abscess location, part of the retina scattered in the bleeding point, part of the blood vessel embolism (A was 3 months after the operation; B was 6 months after the operation).

The patient reported no history of ocular trauma, intravitreal injection, or ocular surgery. A detailed history revealed that the patient had fever and dysuria 5 days prior to presentation. He visited a local hospital and received intravenous fluids and antipyretics for 4 days. Furthermore, apart from urinary tract infection, he had no other risk factors for endogenous endophthalmitis (diabetes, intravenous drug use, indwelling catheter, or immunosuppression). B-scan ultrasonography revealed a dome-shaped subretinal mass with exudative retinal detachment in the left eye (Fig. 2). The fasting blood glucose level was 6.15 mmol/L, white blood cell count was 11.7 × 10^9^/L and C-reactive protein level were 56 mg/L. Urinalysis revealed positive white blood cells (++). Computed tomography and abdominal ultrasonography findings were normal. The body temperature was normal. On the basis of these findings, the patient was diagnosed with endophthalmitis and underwent vitrectomy the following day. Intraoperatively, a subretinal abscess was observed on the nasal side of the optic disc. The abscess was drained via an incision, and silicone oil was filled as a tamponading agent. Vancomycin (1 mg/0.1 mL) and ceftazidime (2.25 mg/0.1 mL) were injected into the vitreous body. A vitreous culture confirmed KP as the causative organism. Therefore, levofloxacin (0.4 g) and ceftriaxone sodium were administered intravenously once a day for 2 weeks, along with levofloxacin and tobramycin eye drops once a day for 1 month. On the second day after the operation, the vitreous cavity was filled with silicone oil when the patient lowered his head, the corneal edema was more severe than before, the anterior chamber empyema, and the remaining peep out (Fig. 1B). Fundus photography at 3 months after the operation, gray-white area for the original abscess location, 6 months after the operation, part of the blood vessel embolism scattered in the bleeding point (Fig. 3). Two weeks after surgery, the best-corrected visual acuity was 0.05, and it improved to 0.1 at 5 months postoperatively.

## 3. Discussion

KP is a Gram-negative opportunistic bacterium that belongs to the Enterobacteriaceae family.^[6]^ It causes a wide range of diseases, including pneumonia, urinary tract infections, bloodstream infections, and sepsis.^[7]^ These infections are particularly problematic among neonates, elderly, and immunocompromised individuals. The co-evolution of KP in response to the challenge of an activated immune system has made it a formidable pathogen that exploits stealth strategies and actively suppresses innate immune defenses to overcome host responses and survive in tissues.^[8]^ In East Asian countries, most cases of endogenous endophthalmitis originating from LA are caused by KP. In recent years, new challenges regarding Kp have emerged, including Hypervirulent Klebsiella pneumoniae (HvKp), which is more virulent than the classical Kp. HvKp has an increased ability to cause central nervous system infections and endophthalmitis, which require rapid recognition and site-specific treatment. HvKp usually infects community-dwelling individuals who are often healthy and induces invasive LA with specific clinical features. Approximately 80% to 90% of cases have LA as the primary focus of infection, followed by renal or lung HvKp infections. Severe visual loss has been reported in 75% of the cases, with 25% showing bilateral involvement.^[9]^ Our patient did not have LA or pulmonary infections, suggesting that a simple severe urinary tract infection can also cause endogenous endophthalmitis.

We reviewed the literature and found 19 studies that explicitly reported endogenous endophthalmitis caused by urinary tract infections^[5,10–27]^ (Table 1). In these cases, >70% of the patients were aged > 50 years and most were men. The sources of urinary-tract infections include urinary calculi, acute pyelonephritis, indwelling catheters, drainage double-J catheters placed during urinary tract surgery, and prostatitis. The pathogen reaches the contents of the eye through the blood, causing endophthalmitis, a rare end-organ disease.

**Table 1:** Summary of the findings of previously published cases of endogenous endophthalmitis caused by urinary tract infection.

Study	Age/sex	Presenting feature	Laboratory diagnosis	Treatment	Risk factors	Species	Outcome
Bouhout et al 2021^[5]^	59/W	Decreased visual acuity Periorbital edema Photophobia Proptosis Pain	Vitreous Blood culture	Ceftazidime (IVI) Moxifloxacin Meropenem (IV) Ciprofloxacin PPV	Diabetes prior bariatric surgery Acute pyelonephritis	KP	Evisceration (R)
Margo et al 1994^[10]^	21y/W	Loss of vision Right conjunctiva injected Anterior chamber cellular reaction Afebrile	Vitreous Blood Urine Sputum culture	Gentamicin (IVI) Vancomycin (IVI) Nafcillin (IV) Ceftazidime (IV) Ciprofloxacin (oral) Vitrectomy	Type I diabetes Urinary tract infection	KP	VA: NLP
Walmsley et al 1996^[11]^	1062y/M	Visual acuity reduced Vitreous infiltrated Vitreous hemorrhage	Urine Blood culture	Ampicillin (IV) Cephalexin (oral) Steroids Ciprofloxacin (IV) PPV	Diabetes Diabetic retinopathy Hypertension Urinary tract infection	*Escherichia coli*	OS: 6/36 OD: NLP
Ang et al 2000^[12]^	81y/W	Redness,pain,swelling Corneal and eyelid edema, Conjunctival injection Left relative afferent pupillary defect Eye movements reduced Anterior chamber cellular reaction Vitritis Afebrile	Vitreous Blood Urine culture	Gentamicin (IVI) Vancomycin (IVI) Ceftriaxone (IV) Cefazolin (IVI) Ciprofloxacin (oral)	Diabetes Ischaemic heart disease	KP	Evisceration (L)
Christensen et al 2004^[13]^	57y/M	Redness Vision reduced Ciliary injection Posterior synechiae Vitreous infiltrate	Blood culture	Ceftazidime (IVI) Vancomycin (IVI) Vitrectomy	Type II diabetes Urinary tract infection	*Staphylococcus aureus*	OD: 0.67 OS:0.02
Toshikuni et al 2006^[14]^	69y/M	Blurred vision Chorioretinal lesions and fluffy Vitreous opacities	Vitreous culture	Fluconazole (oral) Vitrectomy	Urinary tract infection Chronic cystitis Fungemia A double-J stent (L) A bladder catheter	*Candida albicans*	OD: 1.5 OS: 0.6
Hu et al 2007^[15]^	55y/W	Blurred vision Conjunctival hypopyons Vitreous inflammation	Blood Urine culture	Vancomycin (IVI) Ceftazidime (IVI) Ceftriaxone (IV) Gentamicin (IV) Cephalexin	Left ureteroscopy holmium Urinary tract infection	*Pseudomonas spp*	VA: 6/12
Najmi et al 2007^[16]^	83y/M	Vision reduced Diffuse conjunctival injection Corneal endothelial striae Stromal edema Corneal and Vitreous haze Optic nerve head swelling, Preretinal central macular abscess	Urine culture	Fluconazole (oral) Amphotericin B (IVI) Vancomycin (IVI) Amikacin (IVI) PPV	Hypertension Arthritis Urinary tract infections Nephrolithiasis Hydronephrosis Ureteral stent placement	*Candida albicans*	OS: 5/200
Chen et al 2012^[17]^	34y/M	Fever General malaise, Blurred vision	Vitreous Blood Urine culture	Fluconazole (oral) Voriconazole (IVI) PPV	Ureterolithiasis Obstructive hydronephrosis Septic shock	*Yeast C albicans*	OD: 20/20
Sawada et al 2013^[18]^	73y/M	Blurred vision Nausea	Vitreous culture	Vancomycin (IVI) Ceftazidime (IVI) Cefpirome (IV) Imipenem (oral)	Lumbar spinal canal stenosis Prostate surgery Uveitis Secondary glaucoma Acute epididymitis	KP (magA and rmpA genes+)	Enucleation (L)
Cong’En et al 2014^[19]^	69y/W	Periorbital swelling Hypopyon	Vitreous culture	Flucloxacillin Ciprofloxacin Ceftriaxone	Urinary tract infection Type II diabetes Hypertension	*C. koseri*	Enucleation (L)
Lin et al 2002^[20]^	71y/M	Pain Pale conjunctiva Right blind eye Progressive impaired vision Lower grade fever	Vitreous Blood Urine culture	Vancomycin(IVI) Andamikacin(IVI)	Chronic renal insufficiency Steven-Johnson syndrome	KP	Not reported
Tseng et al 1996^[21]^	50y/M	High fever Chills Ocular pain Visual impairment	Urine Blood culture	Amikacin (IVI) Gentamicin (IVI) Cefotaxime	Urinary tract infection Endocarditis	*Escherichia coli*	Die
Bhende et al 2017^[22]^	74y/M	Redness Pain Diminution of vision	Urine Blood culture	Ceftazidime (IVI) Cefotaxime Vancomycin (IVI) Vitreous surgery	Urinary tract infection Septicemia	KP	OS: 3/60
Martel et al 2017^[23]^	60y/M	Redness Visual impairment Non-granulomatous anterior uveitis Vitritis A single subretinal abscess	Urine Blood culture	Fluoroquinolone Ceftazidime (IVI) Vancomycin (IVI) Levofloxacin (oral) Dexamethasone	K. pneumoniae bacteremia Liver abscess Urinary tract infection.	KP	BCVA: 20/20
Dogra et al 2020^[24]^	49y/M	Choroidal neovascular membrane Subretinal hemorrhage Painless vision loss Fever Burning micturition	Blood culture	Piperacillin Tazobactum	Urinary tract infection Chronic liver disease	KP	VA: 6/36
Mohd-Ilham et al 2019^[25]^	39y/W	Anterior chamber inflammation Hypopyon Vitritis Subretinal abscess	Blood culture	Vancomycin (IVI) Ceftazidime (IVI) Ciprofloxacin Cefuroxime (IVI) Vitrectomy with silicone oil Tamponade	Urinary tract infection Pyelonephritis Uncontrolled diabetes	KP	VA: 6/36
Makusha et al 2020^[26]^	89y/M	Decreased vision Hypopyon Diffuse conjunctival chemosis Diffuse vitreous haze Dense vitritis Retinal detachment	Blood Urine Vitreous culture 18F-FDG PET/CT scan	Topical antibiotics Topical steroids	Diabetes Chronic kidney disease Urinary tract infection	*S. marcescens*	Not reported
Khan et al 2019^[27]^	51y/M	Fever Right-sided painless visual loss	Blood Urine Vitreous Wound culture	Intravitreal and intravenous antibiotics.	Prostatic abscess Diabetics Septic pulmonary emboli	*Escherichia coli* KP	Evisceration (R)

IV = intravenous, IVI = intravenous infusion, KP = *Klebsiella pneumonia*.

Nearly half of the patients had diabetes mellitus. Ocular complications are common in patients with diabetes, which can lead to retinal vascular disease. Damage to the blood-ocular barrier can cause vision loss or even blindness. Patients with diabetes have decreased resistance, are prone to infection, and have poor prognosis. Although diabetes control is not significantly associated with the prognosis of endogenous endophthalmitis caused by urinary-tract infections, it remains an important risk factor, and laboratory testing is crucial for identifying the source of infection. Pathogenic bacteria can be detected in blood, urine, and vitreous humor. However, in some patients, infection is not detected in the urine or blood at an early stage of the disease, which causes a delay in diagnosis. Some researchers use 18F-fluorodeoxyglucose-positron emission tomography computed tomography for auxiliary diagnosis; however, its high cost precludes its universal clinical use.^[26]^

In summary, urinary-tract infection is an independent risk factor for endogenous endophthalmitis, which is more common in elderly men. The prognosis is favorable in the early stages. However, when accompanied by other risk factors, the prognosis is poor. Therefore, this case has great significance for exploring the primary pathogenesis and improving the treatment of eye diseases.

## Author contributions

**Data curation:** Cong Ren, Fan Meng, Hao Sun.

**Formal analysis:** Zhongen Li, Bin Guo.

**Funding acquisition:** Bin Guo.

**Investigation:** Cong Ren, Zhongen Li, Fan Meng, Yongle Du.

**Project administration:** Bin Guo.

**Supervision:** Bin Guo.

**Writing – original draft:** Cong Ren, Zhongen Li, Fan Meng, Yongle Du, Hao Sun.

**Writing – review & editing:** Bin Guo.
